# Impact of Improvement in Walking Speed on Hospitalization and Mortality in Females with Cardiovascular Disease

**DOI:** 10.3390/jcm9061755

**Published:** 2020-06-05

**Authors:** Giovanni Grazzi, Gianni Mazzoni, Jonathan Myers, Lorenzo Caruso, Biagio Sassone, Giovanni Pasanisi, Franco Guerzoni, Nicola Napoli, Matteo Pizzolato, Valentina Zerbini, Michele Franchi, Sabrina Masotti, Simona Mandini, Andrea Raisi, Giorgio Chiaranda

**Affiliations:** 1 Center of Sport and Exercise Science, University of Ferrara, 44121 Ferrara, Italy; giovanni.grazzi@unife.it (G.G.); gianni.mazzoni@unife.it (G.M.); lorenzo.caruso@unife.it (L.C.); matteo.pizzolato@unife.it (M.P.); valentina.zerbini@student.unife.it (V.Z.); michele.franchi@unife.it (M.F.); sabrina.masotti@unife.it (S.M.); simona.mandini@unife.it (S.M.); 2 Public Health Department, AUSL Ferrara, 44121 Ferrara, Italy; 3 Veterans Affairs Palo Alto Health Care System, Palo Alto, CA 94304, USA; drj993@aol.com; 4 Stanford Medical School, University of Stanford, Stanford, CA 94305, USA; 5 Department of Morphology, Surgery & Experimental Medicine, University of Ferrara, 44121 Ferrara, Italy; b.sassone@ausl.fe.it; 6 Department of Emergency, Division of Cardiology, Cento SS.ma Annunziata Hospital, 44121 AUSL Ferrara, Italy; 7 Department of Emergency, Division of Cardiology, “Delta” Hospital, AUSL Ferrara, 44121 Ferrara, Italy; g.pasanisi@ausl.fe.it; 8 Health Statistics Unit, University Hospital, 44121 Ferrara, Italy; f.guerzoni@aospfe.it (F.G.); n.napoli@aospfe.it (N.N.); 9 General Directorship for Public Health and Integration Policy, Emilia-Romagna Region, 40133 Bologna, Italy; giorgio.chiaranda@gmail.com; 10 Public Health Department, AUSL Piacenza, 29121 Piacenza, Italy

**Keywords:** women, cardiovascular disease, walking, survival, hospitalization

## Abstract

Cardiovascular disease (CVD) is the principal cause of death in women. Walking speed (WS) is strongly related with mortality and CVD. The rate of all-cause hospitalization or death was assessed in 290 female outpatients with CVD after participation in a cardiac rehabilitation/secondary prevention program (CR/SP) and associated with the WS maintained during a moderate 1 km treadmill-walk. Three-year mortality rates were 57%, 44%, and 29% for the slow (2.1 ± 0.4 km/h), moderate (3.1 ± 0.3 km/h), and fast (4.3 ± 0.6 km/h) walkers, respectively, with adjusted hazard ratios (HRs) of 0.78 (*p =* 0.24) and 0.55 (*p =* 0.03) for moderate and fast walkers compared to the slow walkers. In addition, hospitalization or death was examined four to six years after enrollment as a function of the change in the WS of 176 patients re-assessed during the third year after baseline. The rates of hospitalization or death were higher across tertiles of reduced WS, with 35%, 50%, and 53% for the high (1.5 ± 0.3 km/h), intermediate (0.7 ± 0.2 km/h), and low tertiles (0.2 ± 0.2 km/h). Adjusted HRs were 0.79 (*p =* 0.38) for the intermediate and 0.47 (*p =* 0.02) for the high tertile compared to the low improvement tertile. Improved walking speed was associated with a graded decrease in hospitalization or death from any cause in women undergoing CR/SP.

## 1. Introduction

Despite the current decrease in cardiovascular mortality in women, cardiovascular disease (CVD) continues to be the main cause of death in women [1,2]. Over the last two decades, the American Heart Association, the American College of Cardiology, and the National Heart, Lung and Blood Institute have invested significantly in actions to increase mindfulness of the importance of CVD among women. CVD as the major cause of death among women has been reported to increase from 30% in 1997 to 54% in 2009 [3,4]. Approximately 500,000 women are hospitalized annually for an acute coronary event in the US [3]. Moreover, women have twice the probability of having an adverse outcome after coronary artery bypass graft surgery and are more likely to die within a year after an acute myocardial infarction compared to men [3,4].

The absolute numbers of women living with, and dying of, CVD exceed those of men, as does the number of hospital discharges for heart failure and stroke [5]. In the US, the yearly medical costs of CVD are estimated to increase from USD 396 billion in 2012 to USD 918 billion in 2030. Of these, 60.5% is due to the cost of hospitalization [6]. The prioritization of healthy behaviors (including physical activity), in addition to the optimization of medical treatment of established CVD, is a leading objective of several health organizations, in order to ameliorate cardiovascular health and lessen healthcare costs related to CVD [6].

Referral to cardiac rehabilitation/secondary prevention (CR/SP) is a class I recommendation for women with CVD [3]. Nevertheless, only 15% to 20% of eligible women participate in these programs [3]. A cornerstone of CR/SP is physical activity, with the goal to increase cardiorespiratory fitness and functional ability [3,7,8,9]. Cardiorespiratory fitness (CRF) is a powerful predictor of cardiovascular and all-cause morbidity in subjects with and without CVD [10,11]. CRF has been demonstrated to be strictly related to walking capacity [12,13]. The capability to walk reproduces the combined performance of many organ systems. Walking speed (WS) has been inversely associated with hospitalization, morbidity, and mortality in men and women with CVD [14,15,16]. Slow WS has also been associated with various health-related issues including disability, morbidity, and mortality [17]. Thus, the evaluation of WS is a marker of health and function in aging and disease and has been advocated as a potential additional “vital sign” [18].

WS is a suitable measure of functional capacity, consistent with the daily activities of most adults, and the preferred physical activity by insufficiently active individuals [19]. The WS kept during a moderate and perceptually regulated treadmill-walking test has been shown to be a valid and simple tool for the estimation of CRF [20,21,22,23] and is inversely related to all-cause mortality [24,25,26]. Nevertheless, little is known about the relationship between WS and fatal and non-fatal events in female patients with CVD. In addition, the usefulness and clinical significance of changes in walking capacity over time as predictors of health outcomes is uncertain since the predictive value of WS is mostly founded on a single measure at baseline. Although physical activity behaviors can be modified during a certain follow-up period, interpretations founded on a single measure at baseline can potentially be misleading [25,26].

This study was conducted among women with CVD to examine whether the WS during a moderate 1 km treadmill-walking test is related to hospitalization or death from any cause, and whether variations in WS are accompanied by variations in hospitalization or death.

## 2. Materials and Methods

### 2.1. Study Population

A total of 290 consecutive patients, aged 25–88 years, were referred by their GP to an exercise-based CR/SP program at the Public Health Department and Department of Medicine, Division of Cardiology, AUSL Ferrara, Italy. The main objective of the program was the promotion of a long-term physically active lifestyle in order to improve cardiorespiratory fitness and functional capacity. Patients firstly underwent an inclusive clinical evaluation. Hypertension was defined as blood pressure ≥140/90 mm Hg, or current treatment with antihypertensive drugs. Recent blood chemistry analyses—along with left ventricular ejection fraction derived from an echocardiographic evaluation—were registered. The study protocol has been approved by the Ethics Committee of the University of Ferrara, no. 22–13. An approved written informed consent was obtained from all patients at the time of enrollment.

### 2.2. Clinical Follow-Up

Functional assessment was performed at baseline and habitually during follow-up by administering the 1 km treadmill-walking test (1 km TWT). Details of the protocol have been previously described [20]. Based on the results of the 1 km TWT, patients were educated on how to replicate similar walking sessions at home, progressively increasing the duration, from 20 min up to 60 min per session, at least 2–3 times/week but preferably daily. Participants were identified by the regional Health Service Registry, providing data on date of death and hospitalization. The primary outcome was the composite occurrence of all-cause hospitalization and death ascertained throughout the first three years after baseline. The second outcome was the composite occurrence of hospitalization and death during the subsequent fourth, fifth, and sixth year. Any hospitalization was considered an event. For patients undergoing >1 admission, only the first event was considered in the analysis.

### 2.3. Data Analysis

Medical history was determined from the hospital discharge letter. If >1 cardiovascular diagnosis was recorded during a given re-hospitalization, we defined the diagnosis as follows: A coronary artery bypass graft (CABG) replaced other reasons such as myocardial infarction (MI) or valvular repair/replacement. If the diagnosis was an MI followed by a percutaneous transluminal coronary angioplasty (PTCA), it was classified as an MI. If PTCA was completed without an MI, it was classified as a PTCA. If valvular repair/replacement was performed without an MI, it was classified as a valvular repair/replacement. Other cardiovascular diagnoses such as coronary artery anomalies, heart transplantation, or cardiac tumors were classified as others. Then, patients were grouped into tertiles based on 1) the WS maintained during the 1 km TWT at baseline and 2) the change in WS maintained during the 1 km TWT performed three years later. Differences in continuous variables (age, BMI, LVEF, total and HDL cholesterol, triglycerides, fasting blood glucose) and categorical variables across tertiles were assessed using a one-way analysis of variance and a χ^2^ test for trend, respectively. The covariates considered as potential confounders were age, BMI, family history, fasting glucose, LVEF, hypertension, medical history, serum creatinine, serum triglycerides, smoking status, total and HDL cholesterol, and use of ACE inhibitors, angiotensin receptor blockers, aspirin, β-blockers, calcium antagonists, diuretics, and statins.

To evaluate the association among WS and the occurrence of hospitalization and death over time, we constructed Kaplan–Meier curves. Significantly correlated variables were entered for the fully adjusted multivariable regression model. The composite risk of hospitalization and death was considered independently for each variable, including WS (using increases of 1 km/h); adjustments were made for age. In addition, formal tests of interaction were performed between WS and WS change and all the covariates included in the multivariable models. Patients in the lowest WS tertile at baseline and those in the lowest improvement in the WS tertile three years later were considered the reference groups. The assumption of proportionality for the variables considered in the models was evaluated by the analysis of Schoenfeld residuals. The proportional hazard assumption was met for all models. The level of statistical significance was set at *p* < 0.05. Statistical analyses were performed using MedCalc 17.6 (Ostende, Belgium).

## 3. Results

### 3.1. Baseline Characteristics

A total of 290 subjects completed the 1 km TWT without complications. The average WS resulted in 3.1 ± 1.0 km/h. Table 1 shows the baseline demographics and the clinical characteristics of the study population grouped into tertiles of WS. Comparison between categories shows significant differences for age, triglycerides, history of CABG, MI, use of antihypertensive agents, and diuretics.

### 3.2. Baseline Walking Speed and 3-Year Hospitalization and Death

**Figure 1:**
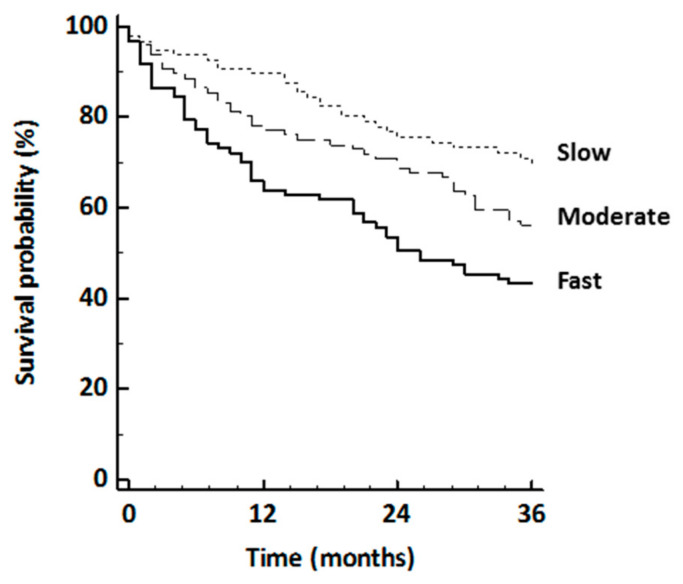
Kaplan–Meier curve showing the rate of hospitalization or death during 36 months after enrolment as a function of walking speed at baseline.

During the 3 years following baseline, 124 subjects (42.7% of the sample) were hospitalized for all causes, and nine died. Among the subjects who died, one, three, and five subjects were in the high, moderate, and low tertiles at baseline, respectively. Because of the limited number of hospitalizations and deaths, the analysis was conducted using a unique all-cause composite outcome [27]. Figure 1 shows the cumulative risk of the composite outcome by tertiles of WS (log rank, *p* < 0.001). Hospitalization or death decreased across increasing tertiles of WS (slowest, *n =* 55, 57%; moderate, *n =* 42, 44%; fastest, *n =* 28, 29%), without interactions between WS and the covariates. The fully adjusted risk for hospitalization or death was lower in the moderate group (hazard ratio (HR) 0.78, 95% CI 0.51 to 1.18, *p =* 0.24) and in the fastest group (HR 0.55, 95% CI 0.32 to 0.93, *p =* 0.03) compared with the slowest. Every 1 km/h increase in walking speed was associated with a fully adjusted 26% reduction in the risk of hospitalization or death (HR 0.74, 95% CI 0.59 to 0.92, *p* < 0.01).

### 3.3. Walking Speed Changes and 4–6 Years’ Hospitalization or Death

**Figure 2:**
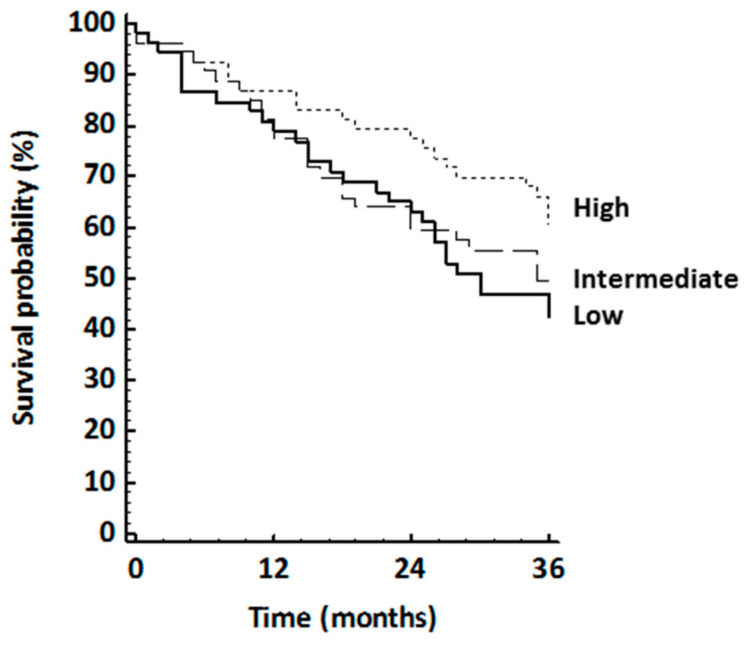
Kaplan–Meier curve showing the rate of hospitalization or death 36 to 72 months after enrolment as a function of walking speed improvement.

During the third-year follow-up, 176 patients were re-evaluated. Among the 105 patients who missed the second test, 36, 33, and 36 subjects were in tertiles one, two, and three at baseline. Compared with the re-evaluated patients, those who missed the second 1 km TWT were less likely to have a history of CABG (35% vs. 45%), were more likely to have a history of PTCA (15% vs. 5%), and had more common use of diuretics (36% vs. 24%). The clinical characteristics of the patients re-evaluated 3 years after baseline are presented in Table 2. WS improved from 3.2 (0.9) to 3.9 (1.0) km/h for the total population. The improvements across tertiles resulted from 3.4 (1.0) to 3.5 (1.0) km/h (*p =* 0.15), from 3.1 (0.9) to 3.8 (0.9) km/h (*p* < 0.0001), and from 3.0 (0.8) to 4.5 (0.9) km/h (*p* < 0.0001) for the low, moderate, and high tertiles, respectively. During 4 and 6 years after baseline, 76 (43% of the sample) subjects were hospitalized and nine died from all causes. The survival curves for hospitalization and death stratified according to tertiles of WS improvement are presented in Figure 2. Hospitalization or death risk was increasingly lower across tertiles of WS improvement and resulted in 53%, 50%, and 35% in the low, intermediate, and high improvement groups (*p* for trend 0.06). There were no significant interactions between WS change and the covariates examined.

Compared with patients in the low improvement tertile, the HRs for those in the moderate improvement and high improvement tertiles were 0.79 (95% CI 0.46 to 1.34, *p =* 0.38) and 0.47 (95% CI 0.25 to 0.88, *p =* 0.02), respectively. Every 1 km/h increase in walking speed was associated with a fully adjusted 42% reduction in the risk of hospitalization or death (HR 0.58, 95% CI 0.39 to 0.87, *p* < 0.01).

## 4. Discussion

In 290 female outpatients with CVD, WS at baseline was inversely associated with the risk of all-cause hospitalization or death, independently from age, clinical history, and established risk factors. During the three years follow-up, patients in the fastest group showed a full adjusted 45% reduction in the risk of hospitalization or death compared to the patients in the slowest group. Correction for baseline WS resulted in a minimal change in these risk estimates. A 26% lower rate of all-cause hospitalization or death was associated with every 1 km/h increase in WS. Similar results have been observed in men and women post-myocardial infarction [28], among men and women with stable ischemic heart disease followed for 8 years [29], among men and women with chronic heart failure followed for one year [30], and in men and women undergoing cardiac surgery [15]. Overall, these studies included 1333 men and 266 women.

The next relevant finding of this study was the inverse association between the improvement in WS and the risk of hospitalization or death documented in 176 women with CVD re-evaluated three years after baseline. Compared to the 59 subjects with a slight change in WS, among the 60 and 61 patients who increased WS by 23% and 50%, reductions of 31% and 53% in hospitalization or death resulted during the fourth to sixth year of follow-up. This strong association persisted after adjustment for confounders. These findings further emphasize the importance of walking capability assessment and counseling, recently recommended as a potential ”vital sign” [18].

Why might being able to walk faster influence hospitalization or death in patients with CVD? Walking is a composite activity, with several factors affecting pace or speed (i.e., cardiorespiratory fitness, neuro-muscular and skeletal condition, habitual physical activity, cognition, sensory and perceptual function, motivation, and mental health) [31,32,33,34]. Therefore, walking speed is considered an indicator of physical fitness and overall health [35].

WS is strongly related to cardiorespiratory fitness [12,13] as well as mortality from various causes [36]. Thus, it is not surprising that the greatest benefits were found in the fastest walkers, perhaps reflecting overall higher levels of physical activity. In fact, regular physical activity favorably influences numerous established CVD risk factors, including fibrinolysis and coagulability, inflammation, and autonomic function, as well as an age-related decline in myocardial blood flow and endothelium-dependent vasodilatation [37,38]. All these factors may contribute to WS and help explain the association between change in WS and all-cause hospitalization or death.

### 4.1. Strengths of the Study

The current study included a group of women with CVD over a wide range of ages and functional capacities, over a long follow-up period. Secondly, the ease of WS makes it simple to be used in clinical practice. Thirdly, in comparison to other common walking tests (often performed at a near maximum exercise intensity), the 1 km TWT is carried out at a moderate effort and is therefore more agreeable to patients and is likely to be safer. Finally, the large inclusion criteria used in this study is likely to reflect real-world clinical practice.

### 4.2. Limitations of the Study

This study was conducted in women with CVD who willingly participated in an exercise-based secondary prevention program; thus, they may not represent the general population. Since the adherence rate was not determined, a causal relationship between the change in physically active behavior and the change in WS cannot be established. Cognitive decline or social, behavioral or psychological factors that could independently affect WS [39,40,41] were not considered. In addition, environmental and cultural factors that could influence WS [31] were not examined. Finally, considering the observational nature of the study, the grade to which walking faster may causally influence hospitalization or death cannot be addressed.

## 5. Conclusions

The present results support the concept that in women with CVD, those being able to walk faster have a lower rate of hospitalization and death, which represent relevant endpoints in clinical and research settings. The change in survival and hospitalization rates is related to the extent of the change in WS. These results propose that the walking speed maintained during a moderate endurance test can be used by health professionals seeking simple tools for the promotion and maintenance of physically active lifestyles in women with cardiovascular disease.

## Figures and Tables

**Table 1:** Demographic and clinical characteristics of the 290 subjects by baseline tertiles of walking speed.

	All Subjects	Slow	Moderate	Fast	*p* Value
***n***	290	97	96	97	
Walking speed (km/h)	3.1 (0.9)	2.1 (0.4)	3.1 (0.3)	4.3 (0.6)	-
Demographics					
Age	65 (10)	71 (7)	65 (10)	60 (10)	<0.001
BMI	26.4 (4.1)	27.2 (5.1)	26.4 (3.9)	25.7 (3.1)	0.06
LV ejection fraction (%)	59 (9)	59 (9)	59 (10)	59 (9)	0.9
Risk factor					
Current smoking (%)	9	1	8	9	0.2
Hypertension (%)	72	75	77	64	0.09
Family history (%)	46	44	38	55	0.06
Fasting glucose (mg/dL)	104 (27)	105 (32)	107 (29)	99 (20)	0.3
Total cholesterol (mg/dL)	211 (49)	214 (58)	216 (49)	204 (39)	0.3
HDL cholesterol (mg/dL)	51 (14)	56 (14)	56 (14)	57 (15)	0.8
Triglycerides (mg/dL)	135 (76)	134 (69)	155 (99)	121 (57)	0.04
Serum creatinine (mg/dL)	0.98 (0.33)	1.01 (0.25)	0.99 (0.46)	0.93 (0.23)	0.4
Medical history (%)					
CABG	40	46	46	26	0.03
Myocardial infarction	18	6	14	34	<0.0001
PTCA	9	6	10	9	0.5
Valvular repair/replacement	29	37	27	22	0.06
Other	2	2	1	4	0.4
Medications (%)					
ACE inhibitor or ARB	53	58	60	40	0.006
Aspirin	63	57	65	68	0.2
β-blockers	52	44	56	57	0.2
Calcium antagonists	15	17	14	12	0.3
Diuretics	28	45	28	11	<0.0001
Statins	46	41	45	51	0.4

Data are presented as mean (standard deviation) or percentage. ACE, angiotensin-converting enzyme; ARB, angiotensin receptor blocker; BMI, Body Mass Index; CABG, Coronary Artery Bypass Graft; LV, Left Ventricular; PTCA, Percutaneous Transluminal Coronary Angioplasty, stenting or both.

**Table 2:** Clinical characteristics of the subjects 3 years after baseline subdivided by tertiles of improvement of walking speed.

	All (*n =* 176)	Low Improvement (*n =* 59)	Moderate Improvement (*n =* 60)	High Improvement (*n =* 57)	*p* Value
Walking speed improvement from baseline (km/h)	0.7 (0.6)	0.1 (0.3)	0.7 (0.2)	1.5 (0.4)	-
General					
Age	65 (9)	66 (10)	65 (8)	63 (9)	0.18
BMI	26.6 (3.8)	27.7 (3.6)	26.4 (4.1)	26.8 (3.8)	0.85
LV ejection fraction (%)	59 (9)	60 (11)	59 (8)	59 (10)	0.88
Risk factor					
Current smoking (%)	4	5	5	2	0.71
Hypertension (%)	76	80	74	72	0.52
Family history (%)	45	38	53	45	0.29
Fasting glucose (mg/dL)	104 (24)	99 (20)	103 (22)	109 (28)	0.28
Total cholesterol (mg/dL)	213 (50)	207 (40)	219 (60)	214 (49)	0.53
HDL cholesterol (mg/dL)	57 (13)	59 (13)	54 (12)	57 (14)	0.32
Triglycerides (mg/dL)	125 (57)	117 (44)	127 (58)	130 (66)	0.62
Serum creatinine (mg/dL)	0.97 (0.3)	0.99 (0.2)	0.95 (0.3)	0.97 (0.3)	0.81
Medical history					
CABG (%)	48	51	42	53	0.46
Myocardial infarction (%)	15	15	19	9	0.39
PTCA (%)	6	6	8	4	0.69
Valvular replacement (%)	29	28	30	28	0.06
Other (%)	1	0	0	2	0.41
Medications					
ACE inhibitor or ARB (%)	52	51	47	58	0.48
Aspirin (%)	63	62	66	60	0.77
β-blockers (%)	52	42	57	57	0.21
Calcium antagonists (%)	18	21	21	11	0.34
Diuretics (%)	24	25	21	26	0.39
Statins (%)	50	49	51	49	0.98

Values are presented as mean (standard deviation, SD) or %. Abbreviations: ACE, angiotensin-converting enzyme; ARB, angiotensin receptor blocker; BMI, Body Mass Index; CABG, Coronary Artery Bypass Graft; HDL, high-density lipoproteins; LV, Left Ventricular; PTCA, Percutaneous Transluminal Coronary Angioplasty, stenting or both. The values of the variables considered (except walking speed) are baseline values.
